# Association between excessive screen time and falls, with additional risk from insufficient sleep duration in children and adolescents, a large cross-sectional study in China

**DOI:** 10.3389/fpubh.2024.1452133

**Published:** 2024-12-06

**Authors:** Runquan Zhang, Haiyuan Zhu, Qin Xiao, Qiqi Wu, Yuqing Jin, Tao Liu, Dan Liu, Chunxia Cui, Xiaomei Dong

**Affiliations:** ^1^Department of Public Health and Preventive Medicine, School of Medicine, Jinan University, Guangzhou, China; ^2^Department of Epidemiology, School of Public Health, Southern Medical University, Guangzhou, China; ^3^Inner Mongolia Autonomous Region Center for Disease Control and Prevention, Huhhot, China

**Keywords:** Nutrition and Health Surveillance of Children and Lactating Mothers, children, adolescents, screen time, sleep duration, falls

## Abstract

**Objective:**

Falls is a major global public health issue that occur in all age groups. However, the association between screen time, sleep duration and falls in children and adolescents remains unclear.

**Methods:**

This study included children and adolescents who participated in the 2017 Nutrition and Health Surveillance of Children and Lactating Mothers in China. Screen time, sleep duration and falls in the past 12 months were assessed using baseline questionnaires completed by the participants. We utilized a multivariate logistic regression model to estimate the association between screen time, sleep duration, and falls in children and adolescents. Stratified analyses and sensitivity analyses were performed using the same modelling strategies.

**Results:**

A total of 564 participants (5.7%) self-reported falls in the past 12 months. Multivariate logistic regression analysis revealed that high screen time (> 2 h per day) was associated with a higher incidence of falls (cOR:1.46, 95% CI: 1.22–1.74, *p* < 0.001). The combination of high screen time and low sleep duration was associated with an increased risk of falls compared to the recommended low screen time and high sleep duration group (cOR: 1.62, 95% CI: 1.25–2.09, *p* < 0.001). After adjusting for relevant covariates, the associations remained significant (aOR: 1.30, 95% CI: 1.08–1.56, *p* = 0.006; aOR: 1.43, 95% CI: 1.10–1.87, *p* = 0.008).

**Conclusion:**

Our study demonstrates that both high screen time and the combination of high screen time and low sleep duration were associated with an increased risk of falls. Interventions to promote healthy physical development should commence in early childhood to decrease the incidence of fall injuries in children and adolescents.

## 1 Introduction

Falls, also known as falling or falling injury, include sliding, tripping, and falling on the same plane, as well as falling from one plane to another (1). Falls is a major global public health issue that occur in all age groups. Globally, falls accounted for 696,000 deaths in 2017, making them the second leading cause of injury-related deaths worldwide (2). In 2015, the total medical costs incurred by children aged 0 to 19 years for emergency department visits due to falls were $6.3 billion, accounting for more than half of the total medical costs of injury-related emergency department visits in the United States (3). Age is a significant risk factor for falls, with the risk of injury and death from falls increasing among older adults (4). Consequently, previous research into falls has predominantly focused on middle-aged and older adult people. However, children and adolescents are also a high-risk group for falling. Falls are a common cause of non-fatal injuries among children and adolescents, and the leading cause of death and disability among children globally (5). Although the incidence of death due to fall injuries is low among children, the incidence of emergency department visits and hospitalizations due to falls is high among pediatric injuries (6). Falls are the leading cause of traumatic brain injury in the United States, with children aged 0 to 14 accounting for approximately one-third of the 1.4 million people who suffer a TBI each year (7). Therefore, it is imperative to identify and control the risk factors associated with falls in children and adolescents.

Screen time refers to the duration spent engaging with electronic or digital media, including mobile phones, tablets, computers, and televisions (8). It is evident that screens are an integral part of modern life in 2024. A significant proportion of children and adolescents now spend more than half of their day engaged with screens. The American Academy of Pediatrics (AAP) and the Physical Activity Guidelines for the Chinese Population (2021) recommend no more than 2 h of screen time per day (9, 10). Previous studies have demonstrated that, prior to the onset of the COVID-19 pandemic, children and adolescents were already exceeding the recommended screen time limit of 2 h per day (11). A literature review indicates that the increase in daily screen time among adolescents worldwide during the COVID-19 pandemic ranged from 55 min to 2.9 h (12). The COVID-19 pandemic has prompted numerous primary and middle schools to adopt online teaching through the internet, accelerating the prevalence of excessive screen time (13). The use of interactive screens in educational settings has the potential to enhance learning outcomes (14). However, prolonged screen use among children and adolescents may result in adverse effects on sensorimotor development, executive function, and attention (15).

There is evidence suggesting that the duration and quality of sleep in children are associated with a wide range of health outcomes (16). The periods of childhood and adolescence are a crucial phase in the development of self-control (17). The ability to regulate one’s sleep–wake cycle is also a vital aspect of this development. The American Academy of Sleep Medicine (AASM) recommends that children aged 6 to 12 years old should sleep for 9 to 12 h per day, while adolescents aged 13 to 18 years old should sleep for 8 to 10 h per day to promote optimal health (18). The Healthy China Initiative (2019–2030) recommends that primary, middle, and high school students should sleep no less than 10, 9 and 8 h a day, respectively (15). Nevertheless, many children and adolescents currently face the problem of low sleep duration (19, 20). A survey of child and adolescent health in the United States revealed varying levels of sleep deprivation among children and adolescents aged 0–17 from 2003 to 2011/2012 (21). Previous studies have demonstrated that insufficient sleep is a significant risk factor for falls and injuries in both older adults and adolescents (16, 22). And heavy screen use has been identified as being associated with shorter sleep duration and longer sleep delays before falling asleep (23). The relationship between screen time and sleep duration may be synergistic or interactive. However, few studies have examined the association between the combinations of these variables and falls in children and adolescents. Therefore, we used a large cross-sectional study to examine the association between the combination of screen time, sleep duration and falls in children and adolescent.

## 2 Materials and methods

### 2.1 Study design and participants

The data for this study were collected from the Chinese National Nutrition and Health Surveillance of Children and Lactating Mothers conducted in 2017. The subjects were selected through multi-stage stratified random sampling, and 125 surveillance sites were identified in 31 provinces (autonomous regions and municipalities) in China and allocated according to population proportion. The number of surveillance points allocated to large cities, small and medium-sized cities, ordinary rural areas and poor rural areas was 5, 57, 50, and 13, respectively. Each surveillance point (city/district/county) was selected from two towns (streets), and each town (street) is selected from two village (residential) committees. A total of at least 280 children and adolescents aged 6 to 17 years were investigated at each surveillance site. This was conducted in 10 classes, with grades 1 to 6, 7 to 8, and 10 to 11. The total number of participants in each class was 28, with 14 males and 14 females. The details of the study design have been explained in an earlier publication (24). The population included in this study was derived from the Chinese National Nutrition and Health Surveillance of Children and Lactating Mothers, conducted in Guangdong, Jiangsu, Shandong, Inner Mongolia Autonomous Region, and Guizhou Province in 2017. This sample was nationally representative.

**Figure 1:**
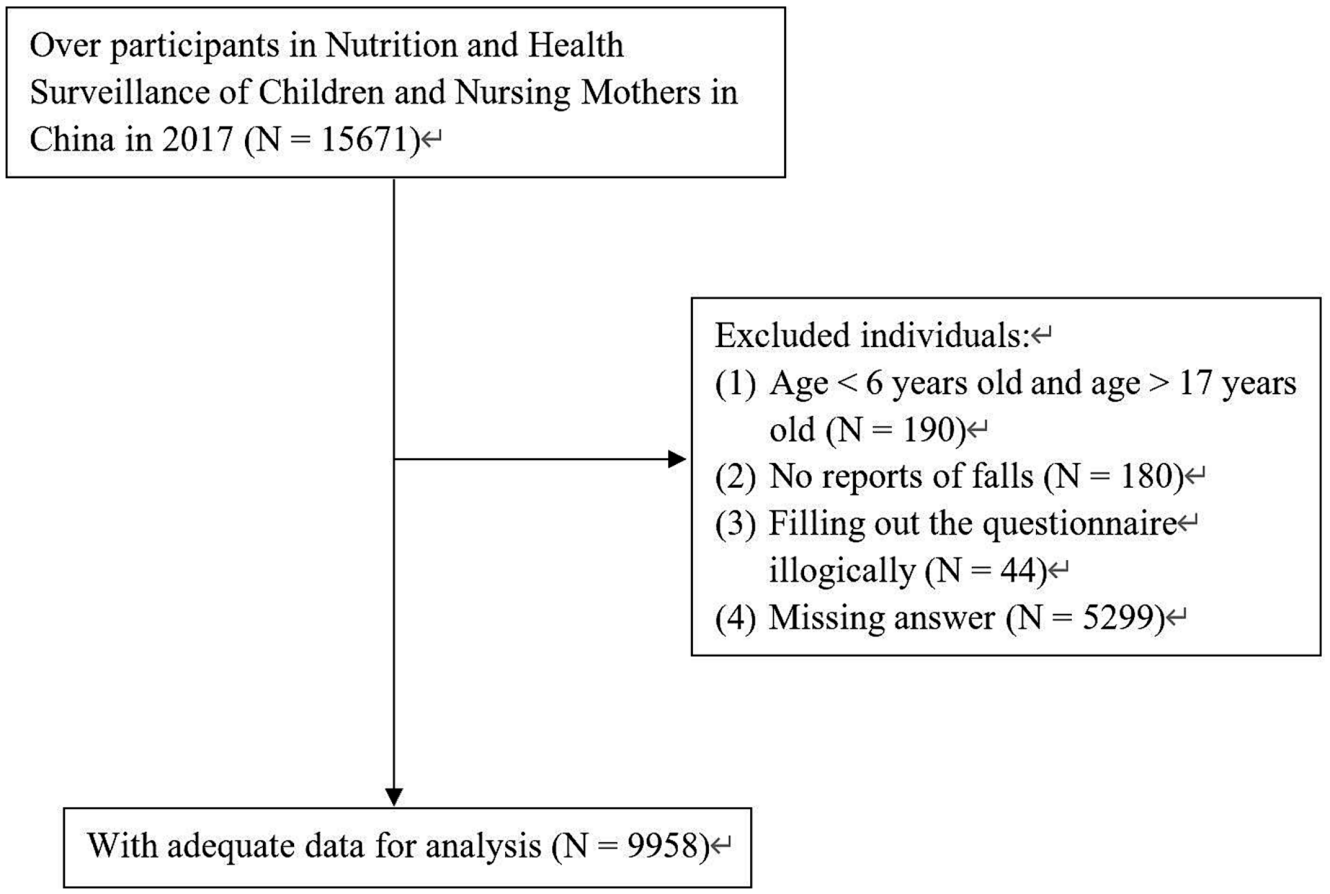
Flow chart of study participant.

To study the incidence of falls in children and adolescents, we limited the participants in this survey to those aged between 6 and 17 years, excluding those younger than 6 years and older than 17 years (*n* = 190). Additionally, we excluded participants in the 2017 survey who did not report whether they had experienced a fall (*n* = 180). Participants with inconsistent logic (*n* = 44) and missing answers (*n* = 5,299) were also excluded. The final study included 9,958 participants (see Figure 1).

### 2.2 Ethical approval and consent to participate

This study was approved by the Ethics Committee of the National Institute of Nutrition and Health for Disease Control and Prevention (No. 201614). Participants could be enrolled in this study only if informed consent was signed by their legal guardian or themselves.

### 2.3 Weighted average screen time

Participants were asked how much time per day they spent sitting, leaning, or lying down doing the following: (a) studying; (b) watching TV or DVDs; (c) using mobile phones; (d) playing video games; (e) using tablets or computers; (f) reading books, magazines and newspapers in their free time; (g) using other electronic screen devices. Screen time was defined as the sum of time spent on (b), (c), (d), (e), and (g). Traditionally, the school day runs from Monday to Friday and the weekend is considered a rest day. Therefore, the data from school days and rest days were weighted by 5/7 and 2/7, respectively, to calculate the weighted average daily screen time for all participants. Weighted average screen time was calculated: (5 × school days screen time +2 × rest days screen time)/7. According to the Physical Activity Guidelines for the Chinese Population (2021) and the standard recommendation of the AAP (10, 25), excessive screen time is defined as more than 2 h per day.

### 2.4 Weighted average sleep duration

Weighted average sleep duration (hours per day) was calculated by asking participants what time they went to bed each night and woke up each morning on school days and rest days. Data from school days and rest days were weighted by 5/7 and 2/7, respectively, to obtain the average daily sleep duration for all participants (26). Weighted average sleep duration was calculated: (5 × school days sleep duration +2 × rest days sleep duration) / 7. The Healthy China Action (2019–2030) recommends that elementary school students, middle school students, and high school students sleep no less than 10, 9, and 8 h per day, respectively (27). Elementary school students sleeping less than 10 h per day, middle school students sleeping less than 9 h per day, and high school students sleeping less than 8 h per day are considered to have insufficient sleep duration.

### 2.5 Falls in children and adolescents

Falls includes sliding, tripping and falling on the same level, and falling from one level to another, such as falling from a height (1). The definition of falls in this study includes those children and adolescents who have been diagnosed with a fall by a medical unit or who have taken a leave of absence for more than half a day due to a fall. Participants self-reported whether they had experienced a fall in the past 12 months. Participants provided binary responses, either ‘no’ or ‘yes’.

### 2.6 Covariates

Covariates included sex, age, father’s education level, mother’s education level, parents were migrant workers, BMI level, medical insurance, fasting blood glucose (FBG) level, difficulty falling asleep, family history of asthma, family history of hypertension, family history of diabetes, and daily moderate-to-vigorous physical activity (MVPA) time. BMI was calculated using height and weight data collected by uniformly trained staff from the Centers for Disease Control and Prevention (CDC) during the body measurement process, and was based on the sex-specific BMI reference thresholds for overweight and obesity screening for school-aged children and adolescents aged 6 to 18 years, as defined in China’s WS/T 586–2018. FBG levels were measured by collecting fasting venous blood samples. The remaining variables were collected using standardized questionnaires provided by the Chinese CDC project team. MVPA is defined as any form of activity that may result in shortness of breath or sweating in the child participant. The MVPA time is self-reported by the study participants. The details of the study design have been explained in an earlier publication (24).

### 2.7 Statistical analysis

Continuous variables are reported as mean ± standard deviation (x̄ ± s), and categorical variables as percentages (n, %). The ANOVA was used for continuous variables, and the chi-square test was used for categorical variables to compare baseline characteristics. A pair-to-pair combination of screen time and sleep duration was established. Group 1 was characterized by low screen time and high sleep duration, Group 2 by low screen time and low sleep duration, Group 3 by high screen time and high sleep duration, and Group 4 by high screen time and low sleep duration. A multivariate logistic regression model was used to estimate the odds ratios (ORs) and 95% confidence intervals (CIs) for screen time, sleep duration, and falls. Model 1 includes a single variable; Model 2 adjusts for age and sex; Model 3 further adjusts all the relevant covariates. Stratified analyses were performed for different sex, age groups (6 to <12 years old, 12 to 17 years old), BMI levels, FBG levels, difficulty falling asleep, and MVPA time to assess potential interactions by adding the interaction terms of the combination of the above grouping factors with screen time and sleep duration to the logistic model. In sensitivity analyses, screen time was further divided into three groups for more detailed analysis (categorized as “≤ 2 h,” “2–4 h” and “>4 h”). All statistical analyses were performed using R software (version 4.3.1) and IBM SPSS Statistics (version 26.0), with a two-tailed *p* value <0.05 considered statistically significant.

## 3 Result

### 3.1 Baseline characteristics

Table 1 presents the basic characteristics of participants in this study, stratified by their experience of falls. The study included 9,958 participants, of which 5,015 males (50.4%) with a mean age of 11.3 ± 3.1 years. Male participants accounted for 50.4% of the total sample, with a higher proportion (60.3%) among those who fell. The mean age of participants who did not fall was slightly lower than that of those who did (11.2 vs. 11.8 years). A total of 564 participants (5.7%) self-reported experiencing a fall within the past 12 months. Generally, participants who spent more than 2 h on screen time had a higher proportion in the fall group (36.2% vs. 28.0%, *p* < 0.001). 381 participants (67.6%) in the fall group had insufficient sleep duration, compared to 6,134 participants (65.3%) in the non-fall group. The combination of high screen time and low sleep duration was high in the falls group (24.1%). Compared to participants who did not fall, those who did fall were more likely to be male, teenagers, and spend significantly more than 2 h in daily MVPA (all *p* values <0.001).

**Table 1:** Basic characteristics of the participants according to falls in this study.

Variables	Total participants (*n* = 9,958)	Non-fall (*n* = 9,394)	Fall (*n* = 564)	*P* value
Male, n (%)	5,015(50.4)	4,675 (49.8)	340 (60.3)	<0.001^***^
Age, Mean ± SD	11.3 ± 3.1	11.2 ± 3.2	11.8 ± 2.9	<0.001^***^
Father’s education, *n* (%)				0.627
Elementary school and below	1871 (18.8)	1760 (18.7)	111 (19.7)	
Junior high school/senior high school/technical secondary school/vocational school	6,452 (64,8)	6,084 (64.8)	368 (65.2)	
Junior college and above	1,635 (16.4)	1,550 (16.5)	85 (15.1)	
Mother’s education, *n* (%)				0.293
Elementary school and below	2,516 (25.3)	2,358 (25.1)	158 (28.0)	
Junior high school/senior high school/technical secondary school/vocational school	5,994 (60.2)	5,665 (60.3)	329 (58.3)	
Junior college and above	1,448 (14.5)	1,371 (14.6)	77 (13.7)	
Parents were migrant workers, *n* (%)				0.817
No	7,193 (72.2)	6,788 (72.3)	405 (71.8)	
Yes	2,765 (27.8)	2,606 (27.7)	159 (28.2)	
BMI grade, *n* (%)				0.700
Normal	7,747 (77.8)	7,312 (77.8)	435 (77.1)	
Overweight	1,268 (12.7)	1,190 (12.7)	78 (13.8)	
Obesity	943 (9.5)	892 (9.5)	51 (9.0)	
Medical insurance, *n* (%)	9,515 (95.6)	8,985 (95.6)	530 (94.0)	0.061
FBG				0.753
Normal	9,713 (97.5)	9,164 (97.6)	549 (97.3)	
IFG/ diabetes	245 (2.5)	230 (2.4)	15 (2.7)	
Screen time, n (%)				<0.001^***^
< 2 h	7,126 (71.6)	6,766 (72.0)	360 (63.8)	
> =2 h	2,832 (28.4)	2,628 (28.0)	204 (36.2)	
Sleep duration, *n* (%)				0.270
Insufficient	3,443 (34.6)	3,260 (34.7)	183 (32.4)	
Sufficient	6,515 (65.4)	6,134 (65.3)	381 (67.6)	
Difficulty falling asleep, *n* (%)				<0.001^***^
No	5,168 (51.9)	4,923 (52.4)	245 (43.4)	
Yes	4,790 (48.1)	4,471 (47.6)	319 (56.6)	
Family history of asthma, *n* (%)				0.796
No	9,486 (95.3)	8,950 (95.3)	536 (95.0)	
Yes	472 (4.7)	444 (4.7)	28 (5.0)	
Family history of hypertension, *n* (%)				0.108
No	6,318 (63.4)	5,978 (63.6)	340 (60.3)	
Yes	3,640 (36.6)	3,416 (36.4)	224 (39.7)	
Family history of diabetes, *n* (%)				0.986
No	8,596 (86.3)	8,109 (86.3)	487 (86.3)	
Yes	1,362 (13.7)	1,285 (13.7)	77 (13.7)	
MVPA daily, *n* (%)				<0.001^***^
< 1 h	5,111 (51.3)	4,861 (51.7)	250 (44.3)	
1 ~ <2 h	3,439 (34.5)	3,236 (34.4)	203 (36.0)	
> =2 h	1,408 (14.1)	1,297 (13.8)	111 (19.7)	
Screen time and sleep duration, *n* (%)				<0.001^***^
Group1 (low screen time; high sleep duration)	2,457 (24.7)	2,342 (24.9)	115 (20.4)	
Group2 (low screen time; low sleep duration)	4,669 (46.9)	4,424 (47.1)	245 (43.4)	
Group3 (high screen time; high sleep duration)	986 (9.9)	918 (9.8)	68 (12.1)	
Group4 (high screen time; low sleep duration)	1,846 (18.5)	1,710 (18.2)	136 (24.1)	

*p < 0.05; **p < 0.01; ***P < 0.001.

Supplementary Table S1 presents the basic characteristics of participants in this study, stratified by their screen time. 56.0% male participants had a higher proportion exceeding the recommended screen time of 2 h. The mean age of participants whose screen time was less than 2 h was found to be slightly lower than that of those whose screen time exceeded 2 h (11.0 vs. 12.1 years). Participants who fall within 12 months had a higher proportion in the high screen time group (7.2% vs. 5.0%, *p* < 0.001). 968 participants (34.8%) in the high screen time group had insufficient sleep duration, compared to 2,457 participants (34.5%) in the low screen time group. However, this difference did not reach statistical significance. In addition, high screen time were more common among male students (56.0%), those with parents who were migrant workers (29.2%), those experiencing sleep difficulties (55.8%), and those with a low daily MVPA.

### 3.2 Association with screen time, sleep duration and falls

Table 2 showed the independent association of the high screen time and an increased risk of falls. In comparison to the recommended low screen time (≤ 2 h per day), high screen time was associated with a higher risk of falls (cOR: 1.46, 95%CI: 1.22–1.74, *p* < 0.001). After adjusting for covariates, high screen time was independently associated with an increased risk of falls (aOR: 1.30, 95%CI: 1.08–1.56, *p* = 0.006).

**Table 2:** Association between screen time, sleep duration and falls in children and adolescents.

Variables	Model 1^a^			Model 2^b^			Model 3^c^		
	OR	95%CI	*P* value	OR	95%CI	*P* value	OR	95%CI	*P* value
Screen time^d^									
≤ 2 h	ref.								
> 2 h	1.46	1.22–1.74	<0.001^***^	1.39	1.16–1.66	<0.001^***^	1.30	1.08–1.56	0.006^**^
Sleep duration^e^									
Sufficient	ref.								
Insufficient	1.11	0.92–1.33	0.274	1.10	0.92–1.32	0.310	1.10	0.91–1.33	0.305

a Model 1 unadjusted.

b Model 2 adjusted for sex and age.

c Model 3 adjusted for sex, age, father’s education, mother’s education, Parents were migrant workers, medical insurance, BMI level, FBG level, difficulty falling asleep, family history of asthma, family history of hypertension, family history of diabetes, and daily MVPA time.

d Sleep duration was also incorporated as an additional covariate in Model 3.

e Screen time was also incorporated as an additional covariate in Model 3.

^*^P < 0.05; ^**^P < 0.01; ^***^P < 0.001.

Furthermore, our study also revealed that the joint association of screen time with sleep duration significantly increased the risk of falls. Table 3 illustrates the independent association between the combination of high screen time and low sleep duration with falls. Compared with the low screen time and high sleep duration group, the combination of high screen time and low sleep duration was associated with an increased risk of falls (cOR: 1.62, 95%CI: 1.25–2.09, *p* < 0.001). After adjusting for covariates, the combination of high screen time and low sleep duration was also found to be independently associated with falls (aOR: 1.43, 95%CI: 1.10–1.87, *p* = 0.008).

**Table 3:** Association between joint association of screen time and sleep duration with falls in children and adolescents.

Variables	Model 1^a^			Model 2^b^			Model 3^c^		
	OR	95%CI	P value	OR	95%CI	P value	OR	95%CI	P value
Group									
Group1(low screen time and high sleep duration)	ref.								
Group2(low screen time and low sleep duration)	1.13	0.90–1.42	0.299	1.13	0.90–1.42	0.300	1.13	0.90–1.43	0.300
Group3(high screen time and high sleep duration)	1.51	1.11–2.06	0.009^**^	1.45	1.06–1.98	0.020^*^	1.36	0.99–1.86	0.056
Group4(high screen time and low sleep duration)	1.62	1.25–2.09	<0.001^***^	1.54	1.18–2.00	0.001^**^	1.43	1.10–1.87	0.008^**^

a Model 1 unadjusted.

b Model 2 adjusted for sex and age.

c Model 3 adjusted for sex, age, father’s education, mother’s education, Parents were migrant workers, medical insurance, BMI level, FBG level, difficulty falling asleep, family history of asthma, family history of hypertension, family history of diabetes, and daily MVPA time.

^*^P < 0.05; ^**^P < 0.01; ^***^P < 0.001.

### 3.3 Stratified analysis

**Figure 2:**
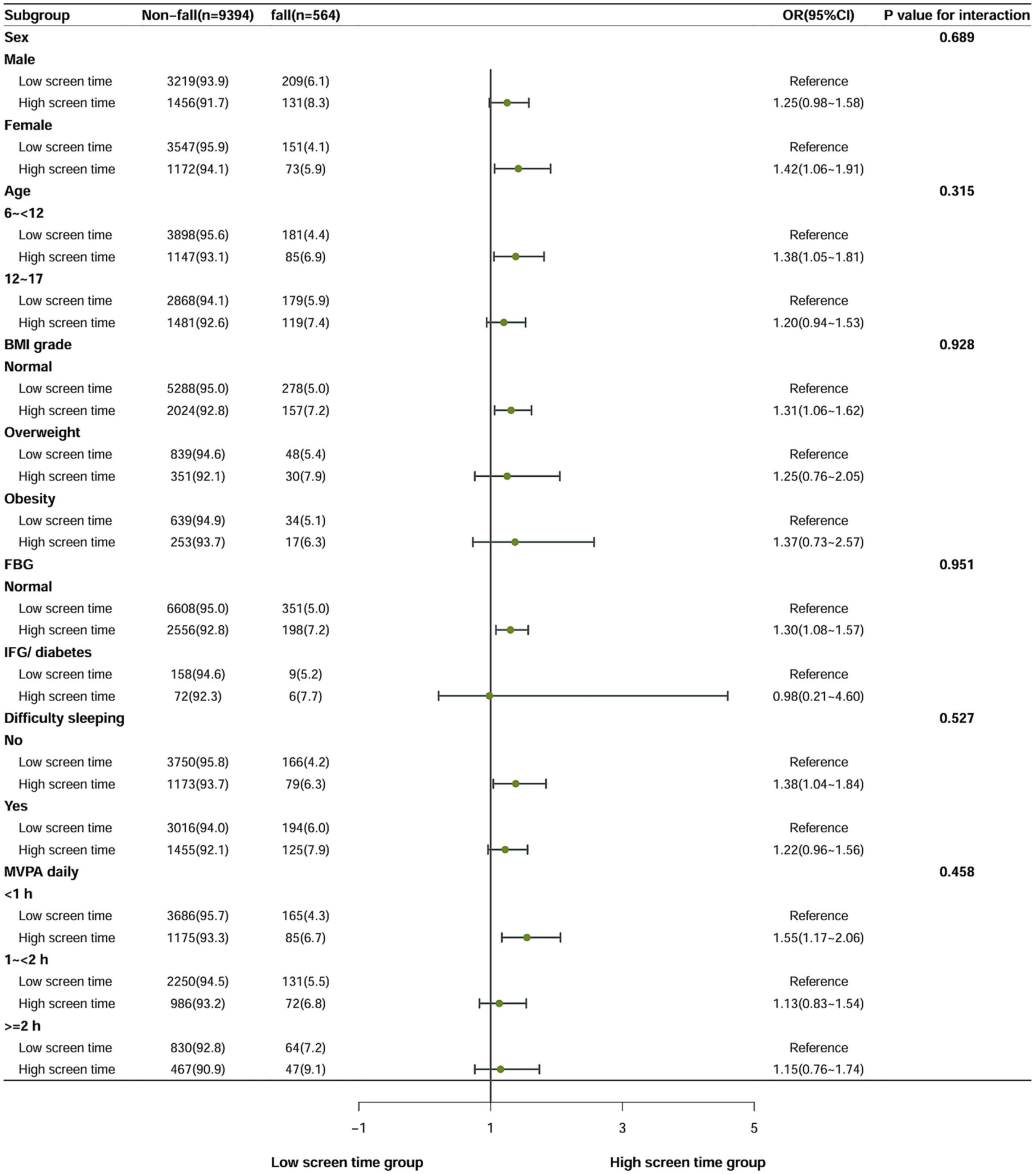
Multivariate-adjusted OR and 95%CI for falls associated with screen time in children and adolescents in stratified analysis (comparing high screen time group with low screen time group).

**Figure 3:**
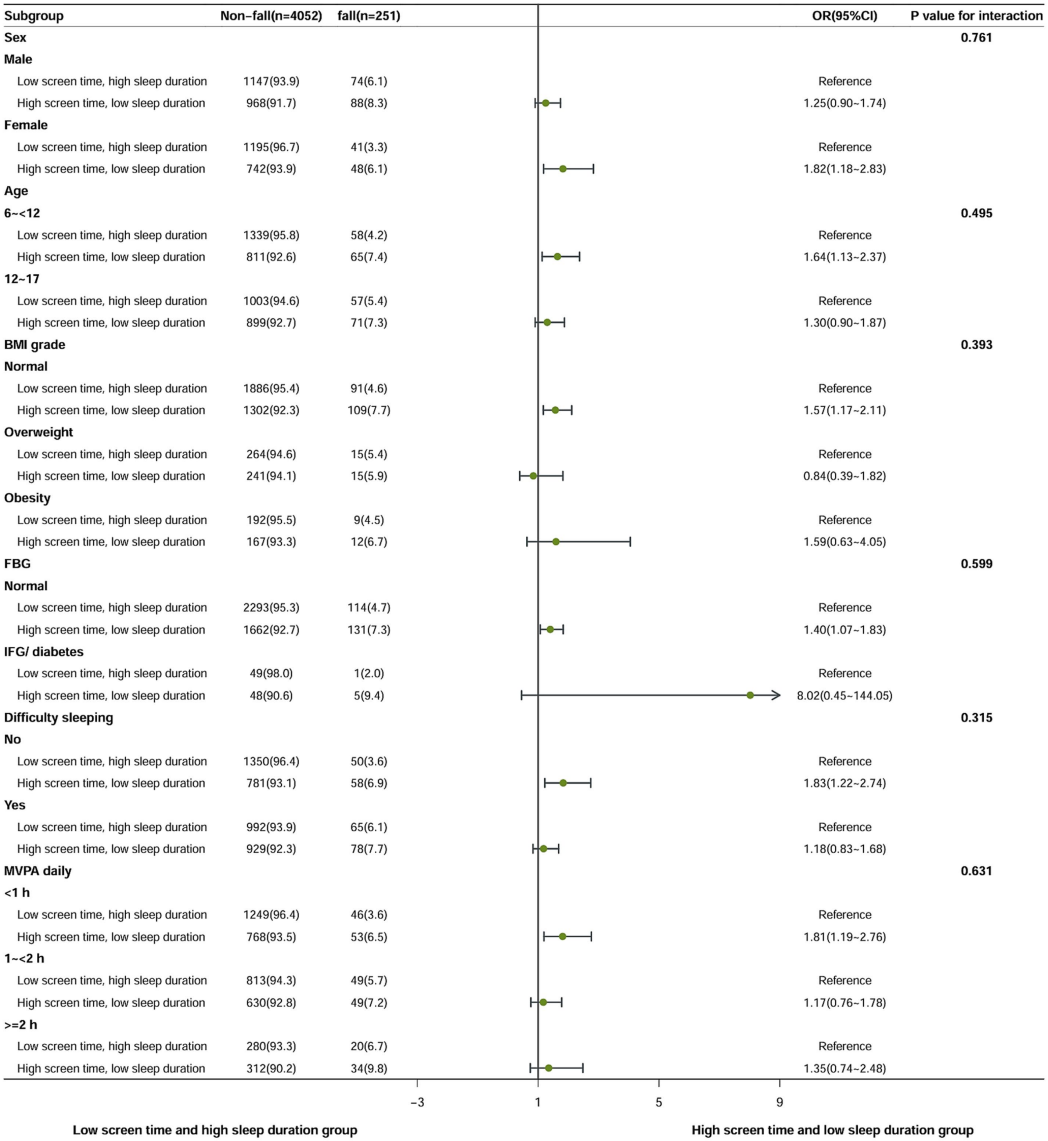
Multivariate-adjusted OR and 95%CI for falls associated with screen time and sleep duration in children and adolescents in stratified analysis (comparing high screen time low sleep duration group with low screen time high sleep duration group).

To further assess the consistency of screen time, sleep duration and falls among different subpopulations, we conducted stratified analysis stratified by sex, age (6- < 12 years old, 12–17 years old), BMI level, FBG level, difficulty falling asleep and MVPA time to further test the stability of the results. Since we observed effects only in persons with high screen time group and high screen time low sleep duration group, we calculated potential effect modifications only for these 2 subgroups. The results of the stratified analysis remained consistent and reliable (as shown in Figures 2, 3). The low screen time group was established as the control group, and it was found that females in the high screen time group exhibited a 42% increased risk of falls (OR = 1.42; 95% CI, 1.06–1.91). The risk of falls in the 6 to <12 age group was 38% higher than the low screen time group(OR = 1.38; 95% CI, 1.05–1.81). Compared with the group with low screen time and high sleep duration, the risk of falls was found to be 82% higher among female in the high screen time and low sleep duration group (OR = 1.82; 95% CI, 1.18–2.83). The 6- < 12 year age group exhibited a 64% higher risk of falls in comparison to the low screen time group (OR = 1.64; 95% CI, 1.13–2.37). There was no interaction between the covariate and the independent variable.

### 3.4 Sensitive analysis

We further divided screen time into three groups (≤ 2 h, 2–4 h, >4 h). We then examined their association with falls in children and adolescents, and the results were basically consistent (see Supplementary Table S2 for details).

## 4 Discussion

Although age is widely acknowledged as a significant risk factor for falls (28), a study conducted in the Netherlands indicates that fall-related injuries represent a significant proportion of unintentional injuries among children and the older adult (29). Falls represent a significant cause of non-fatal injuries in children and adolescents, and are among the leading causes of lifelong disability or death in children (30, 31). The frequent incidence and high emergency department visits indicate that children and adolescents are also at high risk for falls (32, 33). In this study, we used cross-sectional data from the Chinese Nutrition and Health Surveillance of children and Lactating Mothers, conducted in Guangdong, Jiangsu, Shandong, the Inner Mongolia Autonomous Region, and Guizhou provinces. The objective was to examine the association between the combination of screen time, sleep duration, and falls in children and adolescents. The results indicated that, after controlling for relevant covariates, high screen time and the combination of high screen time and low sleep duration were independent risk factors for falls. No interaction was observed between excessive screen time or the combination of high screen time and low sleep duration and sex, age, BMI level, FBG level, difficulty falling asleep, and MVPA time.

Our study indicates that the prevalence of falls among children and adolescents over the past year remains at a considerable level. AlSowailmi et al. (34) found that the incidence of falls among hospitalized children was 9.9 cases per 1,000 patients, which is lower than the 5.7% observed in our study (564/9,958). The reason may be that the incidence of falls in children and adolescents based on hospital case data is lower than that based on community research data, which may not objectively reflect the true incidence of falls in children and adolescents.

It has been suggested that increased screen time may be linked to obesity, poor physical fitness, and other adverse outcomes (13, 35, 36). However, few research has been done on the relationship between excessive screen time and the incidence of injuries such as falls. In this study, nearly 30% of children and adolescents (28.4%) did not adhere to the guideline-recommended amount of screen time, which is generally consistent with the results of previous studies by Chinese scholars (36, 37). In a study of the American children’s health survey (38), the proportion of children who do not meet the recommended screen time guidelines is as high as 67.1%, which may be due to cultural and social differences between China and the United States. Prolonged video viewing can lead to inattention and distraction among children and adolescents (39). Pelicioni et al. (40) demonstrated that prolonged use of mobile phone screens while walking can result in reduced gait stability, impaired postural balance, and an increased risk of accidental falls. Walking on a road necessitates constant awareness of ever-changing road conditions and moving traffic. Some scholars have proposed that an effective solution would be to utilize the sensor in mobile phones to detect walking activity, thereby activating the screen saver and temporarily blocking the screen usage during walking (41).

It is evident that children and adolescents in numerous countries and regions, including China, are currently experiencing insufficient sleep time (19, 21, 42, 43). A lack of sleep may negatively impact their cognitive abilities, reflexes, and self-control (44), resulting in an inability to concentrate and a lack of impulse control. This may, in turn, lead to impaired judgment and an increased risk of falls. Some epidemiological studies have linked insufficient sleep to falls. A population-based cross-sectional study by Kim et al. (45) identified a significant association between sleep duration of ≤5 h per night and falls, with this association being more significant in younger individuals than in older individuals. A case-crossover study demonstrated that sleep duration was shorter in the 24 h before injury (case period) than in the 48 h before injury (control period), and the risk of injury in boys was directly related to sleep deprivation (46). It can be reasonably concluded that sufficient sleep may reduce the incidence of falls. However, our research does not indicate a correlation between a single factor of sleep duration and falls in children and adolescents. One potential explanation for this is that our study categorizes sleep sufficiency and insufficiency in accordance with the Healthy China Action (2019–2030). Another possible reason is that other studies have been more rigorous in defining insufficient sleep duration, including criteria such as less than 5 h per night (16, 45). Therefore, we believe that ensuring adequate sleep duration may effectively reduce the incidence of falls in children and adolescents.

Previous studies using multiple pathway analyses have identified a mediating role for sleep duration in the relationship between early media screen exposure and adverse health outcomes, such as depressive symptoms and attention problems (47, 48). Nuutinen et al. (49) found that screen time in children and adolescents was associated with delayed bedtimes and shorter sleep durations. A US population study found that children and adolescents who spent more time on screens had less sleep time, primarily due to screen use on portable electronic devices (50). One possibility is that as screen time increases, it displaces time that would otherwise be used for sleep, which is known as the time displacement hypothesis (51). Another possibility is that exposure to blue light before bedtime disrupts the sleep–wake cycle of children and adolescents, resulting in delayed melatonin release, a corresponding delay in falling asleep, and a reduction in total sleep time. In addition, children and adolescents have larger pupil diameters than adults, making them more sensitive to the melatonin-inhibiting effects of blue screen light (52). Moderate screen use aims to limit screen time to balance learning and entertainment in children and adolescents. An intervention study by Katie et al. also showed that reducing screen time can increase sleep duration (53). Although there is no direct evidence proving that sleep duration is the mediating link between excessive screen time and falls, the relationship between screen time and sleep duration has empirical implications for injury prevention issues, such as falls.

Stratified analysis indicates that females, children ages 6 to <12 years and MVPA time less than 1 h have a higher risk of falls in the high screen time and high screen time, low sleep duration groups. These findings of our study are consistent with previous research. The effects of excessive screen time are particularly pronounced in younger children, who are more susceptible to its adverse consequences. These effects include, mental health issues, imitate risky behaviors or increased risk-taking during play, and externalizing symptoms of inattention. These may increase the risk of injuries from falls (54–56). A study of adolescents revealed a correlation between higher screen time and lower physical activity duration among female adolescents (57). This finding is also reflected in the time displacement hypothesis. According to the time displacement hypothesis, prolong screen time can displace time allocated to MVPA, which may result in reduced muscle strength and coordination, thereby increasing the risk of injury and falls (51, 58). Higher levels of physical activity are associated with higher skeletal muscle mass index (59). Conversely, children and adolescents who engage in MVPA for less than 1 h may have lower levels of physical activity, which in turn may result in poorer muscle strength and coordination in children and adolescents. Consequently, this may increase the possibility of falls. Although participation in intense MVPA may increase the risk of injury, including falls, a lack of MVPA during growth and insufficient accumulation of muscle strength, particularly in the lower limbs, may also increase the risk of falls (60). It is recommended that children and adolescents adhere to the recommended length of MVPA as set out in the Physical Activity Guidelines, with a focus on injury prevention from falls rather than on reducing activity time (61).

A systematic review indicates that interventions to reduce screen time should commence in early childhood to promote healthy physical development in adolescence (62). Consequently, it is evident that controlling children’s screen time requires parental modeling and involvement (63). A positive correlation was observed between parents’ self-efficacy in regulating their own screen time and the children’s screen time (64). Health promotion programs designed to enhance parents’ awareness and capacity to regulate screen time can help reduce excessive screen time among children and adolescents. Consequently, it is imperative to educate parents and guardians about the potential adverse effects of excessive screen time and to provide strategies to reduce screen time. Furthermore, appropriate bedtimes set by parents can increase the amount of sleep adolescents obtain (34). Parents can set time limits for children and adolescents’ use of electronic devices, or, alternatively, engage in outdoor activities with their children instead of using screens. Health promotion and educational interventions are also extensively utilized in the field of injury prevention, primarily by employing health education methods to enhance individuals’ attitudes, and behaviors, thereby reducing the incidence of injuries (65). It is recommended that educational establishments should be developed and that policies on screen time should be modified in school. Moreover, it may be beneficial for educational institutions to consider implementing time limitations on the use of personal electronic devices in school, with the aim of reducing overall screen time. Schools can also promote the negative effects of excessive screen use and lack of sleep on falls in parent education workshops, school curricula, and community events.

This study examines the relationship between screen time, sleep duration, and falls in children and adolescents, providing a theoretical basis for the prevention and control of fall injuries in this population. However, this study has several limitations. Firstly, the definition of falls in this study includes only those children and adolescents diagnosed by medical units or who had to rest for more than half a day due to falling, excluding those who did not seek medical treatment after falls. This may lead to an underestimation of the incidence of falls. Secondly, screen time, sleep duration, and falls in the past 12 months were self-reported by the participants, which may be subject to recall bias. Additionally, this study is a cross-sectional design, limiting its ability to establish causality. Consequently, it is not possible to draw definitive conclusions about the association between the combination of screen time and sleep duration and falls in children and adolescents. It is recommended that future longitudinal studies verify the causal relationship between different types of screen time and sleep time combinations, including social media, video games, online learning, and both indoor and outdoor falls in children and adolescents.

## 5 Conclusion

The study indicated that high screen time and the combination of high screen time and low sleep duration in children and adolescents were associated with an increased incidence of falls. Therefore, it is recommended that interventions be taken to reduce screen time, ensure adequate sleep duration, and reduce the incidence of falls.

## Data Availability

The original contributions presented in the study are included in the article/Supplementary material, further inquiries can be directed to the corresponding authors.
