# Supplementary material for: Association between excessive screen time and falls, with additional risk from insufficient sleep duration in children and adolescents, a large cross-sectional study in China
Source: Front Public Health. 2024 Dec 6;12:1452133. doi: 10.3389/fpubh.2024.1452133 (PMC11659216; doi:10.3389/fpubh.2024.1452133)
Supplement: Supplementary file 1 [file Table_1.docx]

**Supplementary Table 1.** Basic characteristics of the participants (stratified by screen time)

| Variables | Total participants(n=9958) | Screen time | | P value |
| --- | --- | --- | --- | --- |
|  |  | ≤ 2h (n=7126) | > 2h (n=2832) |  |
| Male, n (%) | 5015(50.4) | 3428 (48.1) | 1587 (56.0) | <0.001^***^ |
| Age, Mean ± SD | 11.3 ± 3.1 | 11.0 ± 3.1 | 12.1 ± 3.2 | <0.001^***^ |
| Father's education, n (%) |  |  |  | <0.001^***^ |
| Elementary school and below | 1871 (18.8) | 1268 (17.8) | 603 (21.3) |  |
| Junior high school/senior high school/technical secondary school/vocational school | 6452 (64,8) | 4578 (64.2) | 1874 (66.2) |  |
| Junior college and above | 1635 (16.4) | 1280 (18.0) | 355 (12.5) |  |
| Mother's education, n (%) |  |  |  | <0.001^***^ |
| Elementary school and below | 2516 (25.3) | 1702 (23.9) | 814 (28.7) |  |
| Junior high school/senior high school/technical secondary school/vocational school | 5994 (60.2) | 4272 (60.0) | 1722 (60.8) |  |
| Junior college and above | 1448 (14.5) | 1152 (16,2) | 296 (10.5) |  |
| Parents were migrant workers, n (%) |  |  |  | 0.044^*^ |
| No | 7193 (72.2) | 5188 (72.8) | 2005 (70.8) |  |
| Yes | 2765 (27.8) | 1938 (27.2) | 827 (29.2) |  |
| BMI grade, n (%) |  |  |  | 0.379 |
| Normal | 7747 (77.8) | 5566 (78.1) | 2181 (77.0) |  |
| Overweight | 1268 (12.7) | 887 (12.4) | 381 (13.5) |  |
| Obesity | 943 (9.5) | 673 (9.4) | 270 (9.5) |  |
| Medical insurance, n (%) | 9515 (95.6) | 6848 (96.1) | 2667 (94.2) | <0.001^***^ |
| FBG |  |  |  | 0.233 |
| Normal | 9713 (97.5) | 6959 (97.7) | 2754 (97.2) |  |
| IFG/ diabetes | 245 (2.5) | 167 (2.3) | 78 (2.8) |  |
| Sleep duration |  |  |  | 0.750 |
| Insufficient | 3443(34.6) | 2457(34.5) | 986(34.8) |  |
| Sufficient | 6515(65.4) | 4669(65.5) | 1846(65.2) |  |
| Difficulty falling asleep, n (%) |  |  |  | <0.001^***^ |
| No | 5168 (51.9) | 3916 (55.0) | 1252 (44.2) |  |
| Yes | 4790 (48.1) | 3210 (45.0) | 1580 (55.8) |  |
| Family history of asthma, n (%) |  |  |  | 0.515 |
| No | 9486 (95.3) | 6782 (95.2) | 2704 (95.5) |  |
| Yes | 472 (4.7) | 344 (4.8) | 128 (4.5) |  |
| Family history of hypertension, n (%) |  |  |  | 0.138 |
| No | 6318 (63.4) | 4489 (63.0) | 1829 (64.6) |  |
| Yes | 3640 (36.6) | 2637 (37.0) | 1003 (35.4) |  |
| Family history of diabetes, n (%) |  |  |  | 0.966 |
| No | 8596 (86.3) | 6152 (86.3) | 2444 (86.3) |  |
| Yes | 1362 (13.7) | 974 (13.7) | 388 (13.7) |  |
| MVPA daily, n (%) |  |  |  | <0.001^***^ |
| <1 h | 5111 (51.3) | 3851 (54.0) | 1260 (44.5) |  |
| 1~<2 h | 3439 (34.5) | 2381 (33.4) | 1058 (37.4) |  |
| >=2 h | 1408 (14.1) | 894 (12.5) | 514 (18.1) |  |
| Fall |  |  |  | <0.001^***^ |
| No | 9394(94.3) | 6766(95.0) | 2628(92.8) |  |
| Yes | 564(5.7) | 360(5.0) | 204(7.2) |  |

*P < 0.05; **P < 0.01; ***P < 0.001

**Supplementary Table 2.** The association between screen time (categorized as “≤ 2 h”, “2-≤ 4 h” and “>4 h”) , sleep duration and falls in children and adolescents.

| **Group** | **Model 1^a^** | | **Model 2^b^** | | **Model 3^c^** | |
| --- | --- | --- | --- | --- | --- | --- |
|  | **OR (95% CI)** | **P value** | **OR (95% CI)** | **P value** | **OR (95% CI)** | **P value** |
| Screen time |  |  |  |  |  |  |
| ≤ 2 h | ref. |  |  |  |  |  |
| 2 -≤ 4 h | 1.40 (1.15-1.70) | 0.001^**^ | 1.34(1.10-1.64) | 0.004^**^ | 1.26(1.03-1.54) | 0.025^*^ |
| > 4 h | 1.66 (1.24-2.23) | 0.001^**^ | 1.55(1.14-2.09) | 0.005^**^ | 1.42(1.05-1.93) | 0.024^*^ |
| Group1(≤ 2 h screen time and high sleep duration) | ref. |  |  |  |  |  |
| Group2(2-≤ 4 h screen time and high sleep duration) | 1.13(0.90-1.42) | 0.299 | 1.13(0.90-1.42) | 0.297 | 1.13(0.90-1.43) | 0.029 |
| Group3(>4 h screen time and high sleep duration) | 1.37(0.90-2.09) | 0.139 | 1.34(0.88-2.05) | 0.173 | 1.27(0.83-1.94) | 0.268 |
| Group4(≤ 2 h screen time and low sleep duration) | 1.48(1.09-2.02) | 0.013^*^ | 1.45(1.06-1.98) | 0.022^*^ | 1.37(1.00-1.88) | 0.052 |
| Group5(2-≤ 4 h screen time and low sleep duration) | 1.63(1.12-2.37) | 0.011^*^ | 1.55(1.06-2.27) | 0.025^*^ | 1.44(0.98-2.12) | 0.064 |
| Group6(>4 h screen time and low sleep duration) | 1.78(1.31-2.42) | <0.001^***^ | 1.64(1.20-2.26) | 0.002^**^ | 1.51(1.10-2.08) | 0.012^*^ |

a: Model 1 unadjusted;

b: Model 2 adjusted for sex and age;

c: Model 3 adjusted for sex, age, father’s education, mother’s education, Parents were migrant workers, medical insurance, BMI level, FBG level, sleep duration, difficulty falling asleep, family history of asthma, family history of hypertension, family history of diabetes, and daily MVPA time.

^*^P < 0.05; ^**^P < 0.01; ^***^P < 0.001
